# Application of Recycled Ultrafiltration Membranes in an Aerobic Membrane Bioreactor (aMBR): A Validation Study

**DOI:** 10.3390/membranes14070149

**Published:** 2024-07-05

**Authors:** Laura Rodríguez-Sáez, Junkal Landaburu-Aguirre, Eloy García-Calvo, Serena Molina

**Affiliations:** 1 IMDEA Water Institute, Av. Punto Com, 2, Alcalá de Henares, 28805 Madrid, Spain; 2 Chemical Engineering Department, Alcalá University, Alcalá de Henares, 28805 Madrid, Spain

**Keywords:** membrane bioreactor (MBR), end-of-life membrane, surface modification, antibiofouling, recycled ultrafiltration membrane, recycling

## Abstract

A validation study using recycled ultrafiltration membranes (r-UF) on an aerobic membrane bioreactor (aMBR) was conducted for the first time. Four different polyethersulfone (PES) membranes were tested using synthetic urban wastewater (COD 0.4–0.5 g/L) during two experimental periods: (i) recycled ultrafiltration membrane (r-UF) and commercial UF membrane (molecular weight cut-off (MWCO) 150 kDa) (c-150 kDa); (ii) r-UF membrane modified by dip-coating using catechol (CA) and polyethyleneimine (PEI) (mr-UF) and c-20 kDa membrane. Permeability, fouling behavior, and permeate quality were evaluated. Extensive membrane characterization was conducted using scanning electron microscopy (SEM), atomic force microscopy (AFM), energy-dispersive X-ray (EDX), and confocal laser scanning microscopy (CLSM). Permeate quality for r-UF and mr-UF membranes was excellent and comparable to that obtained using commercial membranes under similar conditions. Additionally, r-UF and mr-UF membranes presented a steadier performance time. Additionally, r-UF membrane demonstrated less tendency to be fouled (R_f_, m^−1^) r-UF 7.92 ± 0.57 × 10^12^; mr-UF 9.90 ± 0.14 × 10^12^, c-150 kDa 1.56 ± 0.07 × 10^13^ and c-20 kDa 1.25 ± 0.50 × 10^13^. The r-UF membrane showed an excellent antibiofouling character. Therefore, r-UF membranes can be successfully implemented for wastewater treatment in aMBR, being a sustainable and cost-effective alternative to commercial membranes that can contribute to overcome membrane fouling and membrane replacement issues.

## 1. Introduction

Membrane technology has been successfully used and improved for wastewater treatment and many other technological, industrial, and environmental processes for many years now. Particularly, membrane bioreactors (MBRs) for wastewater treatment have acquired great importance due to their great removal/recovery efficiency and lower space requirements compared to conventional activated sludge (CAS) systems resulting from the use of membranes. The installed MBR capacity in 2019 was estimated at over 20 gigalitres per day [1]. Economically speaking, the global market of MBRs is expected to reach EUR 4.9 billion by 2026 [2]. However, MBR processes also present some drawbacks, such as membrane fouling that leads to large chemical consumption; therefore, there is a need for replacement membranes, which substantially increase MBRs’ operational costs [3,4]. The rate of membrane replacement in membrane bioreactor facilities is generally estimated to be between 5 to 10 years [5]. This is why, in recent years, several studies have focused on producing cost-effective membrane alternatives and membranes with antifouling properties. Among them, there is a promising trend of studies based on recycled ultrafiltration membranes (r-UF) that come from end-of-life reverse osmosis membranes (EoL-RO), developed as a viable alternative [6,7]. According to Salinas et al., around 30,000 tons of EoL-RO membranes will be generated and placed into landfills worldwide by 2025, a situation that contradicts the European Union’s principles of the circular economy [8,9,10]. Furthermore, the European Commission is currently working on what it has called the European Green Deal, developing several policies with the main goal of making Europe the first climate-neutral continent by 2050. Among others, the European Green Deal includes as priorities the reduction of water pollution, encouraging water reuse, and improving waste management by extracting as many high-quality resources from waste as possible (thus avoiding its final disposal on landfills and moving towards a circular economy) [11,12,13]. All things considered, the direct and indirect recycling of EoL-RO membrane into r-UF membranes could be an excellent alternative method of reducing resource extraction and reducing waste production [14,15]. With the aim of increasing the lifespan of the membranes and consequently delaying to some extent the landfill disposal of membranes, there are also numerous ongoing studies focused on the evaluation of novel low-cost membranes and membrane surface modification methods (e.g., [16,17]). Furthermore, a novel study of antibiofouling surface modification of the r-UF via a dip-coating process using catechol (CA) and polyethyleneimine (PEI) and using the statistical design of experiments has also been developed [18]. This study demonstrated that the interactions between factors during the surface modification process were as significant as the main factors themselves (e.g., temperature, reaction time, and chemical concentration). Moreover, this study showed that the modification of an r-UF membrane in mild conditions reached a good compromise between performance improvement and energy and chemical consumption. Additionally, the authors concluded that this modification combined with the use of recycled membranes could be a compelling alternative to systems that have a high membrane replacement rate, such us MBR systems. Among the current studies on enhancing membrane-fouling properties, several have been carried out specifically in MBRs. Ashania et al. conducted a long-term evaluation (30 days) of antibiofouling PVDF/Ag-SiO_2_ nanocomposite membranes used in a submerged MBR. Their work concluded that the prepared membranes presented higher antibiofouling properties than the membrane polymer itself [19]. Zhao et al. modified PVDF membranes using graphene oxide for a long-term test (80 days) in an MBR system. Their study concluded that due to the improvement in hydrophilicity, the modified MBR membranes were more resistant to EPS accumulation [17]. Regarding recycled membranes, more recently, a novel proof-of-concept study was developed to assess the performance of r-UF membranes used in an aerobic membrane bioreactor system (aMBR) [20]. According to Rodríguez-Sáez et al., r-UF membranes can be considered an alternative for use in aMBR systems. The r-UF membrane tested in this study showed a lower rate of decline in permeability and similar fouling mechanisms to the commercial microfiltration (MF) membrane tested in the experiment and exhibited excellent results regarding permeate quality. Still, the authors concluded that longer experiments to validate the use or r-UF membranes in aMBRs should be conducted. Up to now, to the best of the authors’ knowledge, validation experiments using r-UF membranes and modified recycled UF membranes (mr-UF) for use in aMBRs has not been performed yet.

The present work entails a validation study aiming to assess the viability of r-UF and modified r-UF obtained from EoL-RO membranes, which were used as submerged flat-sheet membranes in an aMBR system. The performance of the r-UF and mr-UF membranes was compared to the performance of two different commercial membranes commonly used in MBR systems that present very similar characteristics to the r-UF membranes. Four membranes were also tested simultaneously in pairs, whilst maintaining similar working conditions. The process was evaluated in terms of (i) membrane permeability, (ii) resulting permeate quality, and (iii) membrane-fouling behavior.

## 2. Materials and Methods

### 2.1. Experimental Set-Up

**Figure 1:**
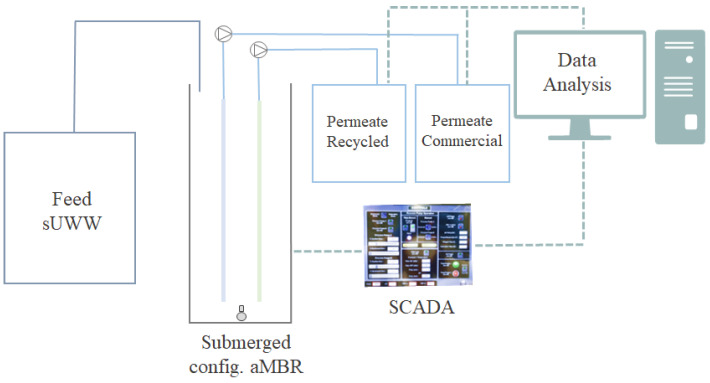
MBR system outline.

A laboratory-scale aMBR (tank capacity of 20 L) was used. Two flat-sheet membrane cartridges (with an effective membrane area of 0.11 m^2^ per membrane) were placed simultaneously in a submerged configuration (Figure 1).

Four different membranes (see Section 2.4) were tested in two different periods: Period I and Period II. Membranes were tested using synthetic wastewater simulating urban wastewater (uSWW) (Table 1).

The primary sludge inoculum was obtained from the Municipal Wastewater Treatment Plant of Guadalajara. To rate the MBR performance, trans-membrane pressure (TMP), pH, and temperature data were monitored constantly. MBR feed and permeate from both membranes in use in every period were sampled and analyzed twice a week. Characterization of the mixed liquor was also performed. Mixed-liquor-suspended solids (MLSS) were determined using UNE-EN 872 [21], BOD_5_ was determined using UNE-EN ISO 9408 [22], COD was determined based on UNE 77004 [23], and TN and TP were determined photometrically (SpectroQuant Pharo 100, Merk) based on UNE-EN ISO 6878 for TP and UNE-EN 25663 for TN [24,25]. DIN 38405 D9 (N-NO_3_) and UNE-EN ISO 6878 (P-PO_4_) methods were used after sample digestion [26,27]. TOC was determined using the TOC-V CSH ASI-V model (Shimadzu) based on standard methods for the examination of water and wastewater. Alkalinity 2320 B. *E. coli* and total coliform were detected and enumerated following a membrane filtration technique based on UNE-EN ISO 9308-1 using chromogenic culture medium CHROMagar™ CCA (Scharlau) [28].

Constant flux (J; 16 L·m^−2^·h^−1^) operation and variable TMP were selected to assess the membrane performance. Membranes operated on cycles of 8 min of suction followed by 2 min of relaxation. After a start-up period in which the biomass was acclimatized to the operating conditions, the laboratory-scale aMBR plant operated in two different experimental periods. During Period I, the membranes used were as follows: (Ia) c-150 kDa (96 days; 16 L·m^−2^·h^−1^) and (Ib) r-UF (96 days; 16 L·m^−2^·h^−1^). During Period II, the membranes used were as follows: (IIa) c-20 kDa (64 days; 16 L·m^−2^·h^−1^) and (IIb) mr-UF (64 days; 16 L·m^−2^·h^−1^). Due to experimental planning on the aMBR system, Period II needed to be limited to 64 days. The laboratory-scale aMBR unit operated at a hydraulic retention time (HRT) of 15 h. For Period I, apart from the samples necessary for analyses and monitoring, 1000 mL/d of mixed liquor (sludge retention time (SRT) ~20 days) was wasted. The MLSS during Period I was approx. 10.64 g/L ± 1.12 g/L; the MLSS during Period II was approx. 7.24 g/L ± 1.69 g/L. Regarding Period II, except for the samples necessary for analyses and monitoring, no biomass was wasted from the reactor (resulting in a sludge retention time, or SRT, of ~∞). Throughout both periods, membrane mechanical cleaning was conducted whenever a sudden pressure spike was observed and/or some problem occurred during the experimental period; otherwise, it was carried out approximately every 20–28 days. Additionally, one chemical cleaning was conducted after the first sudden pressure spike in every experiment (with approx. 1% NaOCl solution for 30 min). Membrane fouling was analyzed at the end of each membrane’s operating period (i.e., after stages Ib and IIb) using a resistance-in-series model proposed by Di Bella et al. to assess the relative importance of pore blocking and cake layer formation to the studied membranes [29,30]. Additionally, additional membrane coupons of every studied membrane were placed into the tank for the experimental period to study the biofouling attached to the membrane at different experimental times: (i) one week; (ii) two weeks; (iii) one month; and (iv) two months.

### 2.2. Membrane Fouling Analysis

Membrane fouling analysis was conducted at the end of both periods, applying the resistance-in-series model suggested by Di Bella et al. in order to measure the importance of the different fouling mechanisms affecting membranes [29,30]. The analysis was conducted following the exact procedure shown in the previous proof-of-concept study [20].

### 2.3. Chemicals

The chemicals consumed along this study were sodium hypochlorite (NaClO 10% *w*/*v*); ethanol (96% EPR Ph.Eur. LABKEM, Barcelona, Spain); polyethylenimine, branched (average Mw 800 by LS, average MN 600 by GPG; 1,2-dihydroxybenzene (ReagentPlus^®^, ≥99%. Merck Life Science, Darmstadt, Germany, S.L.U); Trizma hydrochloride (reagent grade ≥ 99.0%; Trizma base (reagent grade ≥ 99.9%; primary standard and buffer; glucose (C_6_H_12_O_6_) D(+) glucose anhydrous, extra-pure, Ph Eur, BP, USP); meat peptone; urea (reagent-grade urea ACS; sodium chloride (reagent-grade NaCl, ACS, ISO, Reag. Ph Eur); sodium bicarbonate (NaHCO_3_, extra-pure, Pharmpure^®^, Ph Eur, BP, USP); di-potassium hydrogen phosphate, anhydrous (K_2_HPO_4_ for analysis, ExpertQ^®^, ACS, Reag. Ph Eur); calcium chloride dihydrate (CaCl_2_.2H_2_O powder, for analysis via ExpertQ^®^, ACS); magnesium sulfate heptahydrate (MgSO_4_.7H_2_O for analysis via ExpertQ^®^, ACS, Reag. Ph Eur); iron (III) chloride hexahydrate (FeCl_3_.6H_2_O ACS reagent, 97%); pyridine (C_5_H_5_N ExpertQ^®^, ACS, Reag. Ph Eur were purchased from Merck Life Science, Darmstadt, Germany, S.L.U. Sodium hydroxide (reagent-grade NaOH, ACS, Iso, Reag. PhEUr) were purchased from Scharlab S.L., Barcelona, Spain. The ultrapure water (Milli-Q) used in the experiments was obtained from Millipore, Molsheim, France, equipment (conductivity less than 0.055 µS cm^−1^).

### 2.4. Membrane Characterization

Extensive membrane surface characterization was also conducted. Scanning electron microscopy (SEM) combined with energy-dispersive X-ray (EDX) spectroscopy using an S-8000 Model (Hitachi) image device was employed to inspect the surface of the membranes and their elemental composition. Membrane surface roughness was examined by atomic force microscopy (AFM) using a Multimode topographical AFM (Vecco Instruments, Santa Barbara, CA, USA) equipped with a Nanoscope Iva control system (software version 6.14r1). Silicon tapping probes (RTESP, Veeco) were used with a resonance frequency of ~300 kHz and a scan rate of 0.5 Hz; 2 × 2 μm^2^ AFM images were taken for each sample. The confocal biofilm images were obtained under a confocal laser scanning microscope (CLSM Leica SP5, Leica Microsystems). Surface wettability was analyzed using a sessile drop static water contact angle (WCA) using an optical contact angle measurement system (KSV Cam 200 Instrument, Helsinki, Finland). A live/dead backlight bacterial viability kit (Molecular Probes™) was used to observe bacterial accumulation over the membrane surface. To assess if the membranes had been in contact with halogenated compounds, a Fujiwara test was conducted.

### 2.5. Membranes

For the present study, two recycled and two commercial UF membranes (c-20 kDa and c-150 kDa) were evaluated in terms of (i) membrane permeability, (ii) permeate quality, and (iii) membrane-fouling behavior. All the studied membranes’ properties are presented in Table 2. A previous membrane characterization was conducted to assess the properties of the membranes. 

Coupons (0.06 m^2^ area) from four different membranes were used: (i) r-UF membrane model TM720-400 (Toray, Tokyo, Japan); (ii) mr-UF membrane model TM720-400 (Toray, Tokyo, Japan) modified using CA and PEI; (iii) c-20 kDa membrane (Microdyn) model Nadir UP020 20 kDa; and (iv) c-150 kDa membrane (Microdyn, Wiesbaden, Germany) model Nadir UP150 150 kDa. The r-UF membranes were obtained by removing the polyamide layer of EoL-RO membranes by means of exposure to a NaClO dose of 800,000 ppm·h [18]. Adapting the work of Rodríguez-Sáez et al., a scaled-up mr-UF membrane was prepared using a dip-coating process applying the optimized modification conditions established in the previous research (1 h; 30 °C; CA–PEI ratio 1:1) [18]. Additionally, two commercial membranes were selected as a control to set the performance standards.

## 3. Results and Discussion

### 3.1. Membrane Performance

**Figure 2:**
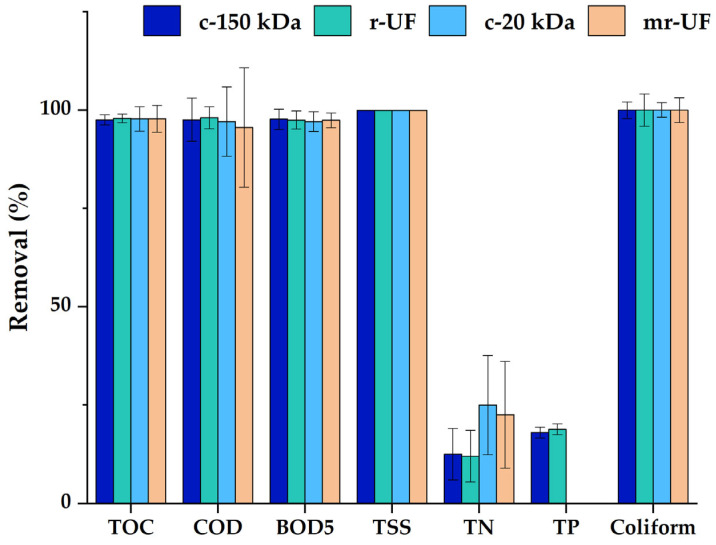
Membrane removal efficiency (R%) for every analyzed parameter of each membrane’s permeate.

Membrane performance was studied by monitoring TMP and permeate quality over the course of the whole experiment. Regarding permeates, overall results showed that the quality obtained using the r-UF and mr-UF membranes was high, and such permeates were also similar to the permeate obtained with the studied commercial membranes. Figure 2 shows the removal efficiency (%) for every membrane during each experimental period. 

**Figure 3:**
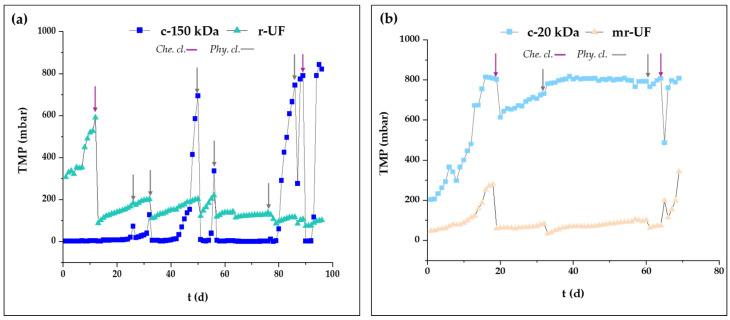
TMP (mbar) for every membrane: (**a**) Period I; (**b**) Period II.

Reduction percentages for all studied membranes were above 99.9% in terms of suspended solids and Coliform bacteria. Additionally, every membrane achieved a reduction of above 97.0% for BOD_5_. For COD, all membranes exceeded 95% reduction, with r-UF and c-150 kDa obtaining the best results. Further, TOC reduction remained above 95% for all the membranes, but the r-UF membrane obtained the best results (98.3%). Figure 3 shows the membrane behavior in terms of TMP over time for experimental Period I and Period II.

The r-UF membrane presented an increase in TMP and therefore a decline in permeability, but after day 11, performances remained quite steady. Moreover, considering the entire experimental period, the general tendency for both r-UF and mr-UF membranes was rather similar, presenting an increase in permeability that was maintained until the end of the experiment. Regarding the c-20 kDa membrane, after the first significant TMP increase, it showed a steady TMP until the end of the experiment. It is possible that this membrane suffered from some grade of irreversible fouling in pore blocking form given its lower MWCO. On the other hand, the c-150 kDa membrane presented a quite low and stable TMP until day 48 when it exhibited a first sharp TMP increase. For the rest of the experiment, the c-150 kDa membrane presented very unstable performance. This is in concordance with the behavior observed by He et al. for PES membranes between 20–70 kDa, membranes with a lower MWCO that showed a faster decline in permeability in the early stages of the experimentation period [31]. However, this tendency changes with time in experiments; significant flux variations have been observed throughout the later stages of membrane filtration with higher MWCO [21]. 

Nonetheless, a number of factors might be considered for deeper understanding of membrane-fouling mechanisms that affect membrane permeability, such us hydrophilicity and surface roughness [31,32]. In the present study, membrane wettability was rather similar for all studied membranes. This was unexpected, especially considering the mr-UF membrane, where lower contact angle values were expected due to the modification process conducted on the membrane surface. However, in order to not compromise other membrane properties (e.g., permeability and roughness) a light modification was selected [18], achieving a null improvement in the hydrophilic character of the modified membrane. 

**Figure 4:**
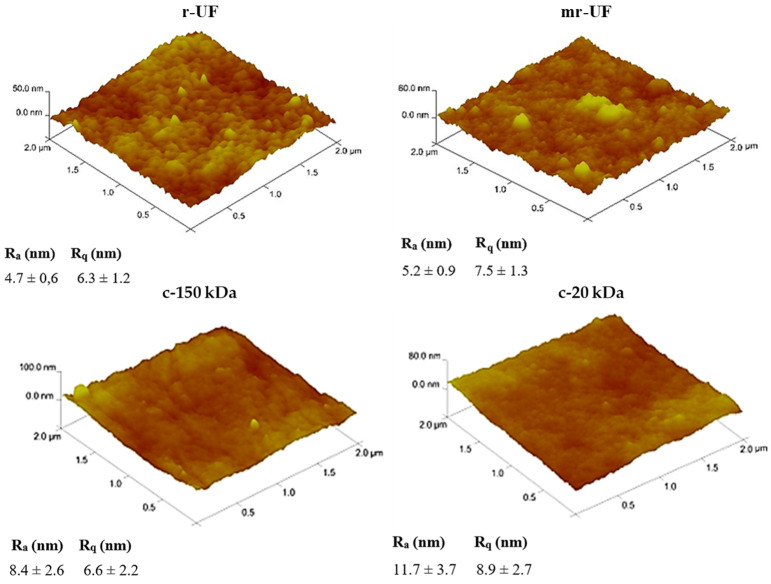
AFM images (2 × 2 μm^2^) for pristine membranes.

Figure 4 shows AFM images and roughness values for every membrane. The r-UF membrane showed the lowest roughness value (Ra = 4.7 ± 0.6 nm; Rq = 6.3 ± 1.2 nm) [17], follower by the mr-UF membrane (Ra = 5.2 ± 0.9 nm; Rq = 7.5 ± 1.3 nm) [17]. On the other hand, the c-20 kDa membrane presented the highest roughness values (Ra = 11.7 ± 3.7 nm; Rq = 8.9 ± 2.7 nm). 

Membranes with higher surface roughness tend to be more prone to developing fouling issues that enhance their decline in permeability [33]. Moreover, membranes that have higher roughness values tend to respond worse to both physical and chemical maintenance cleanings. This leads to worse performance compared to membranes with lower surface roughness in long-term experiments [31]. The same behavior was observed in this study, where for both experimental periods (I and II), the commercial membranes had the highest surface roughness and greater long-term decline in permeability compared to the r-UF and mr-UF membranes.

### 3.2. Membrane Resistance in Series (RIS) Analysis

**Figure 5:**
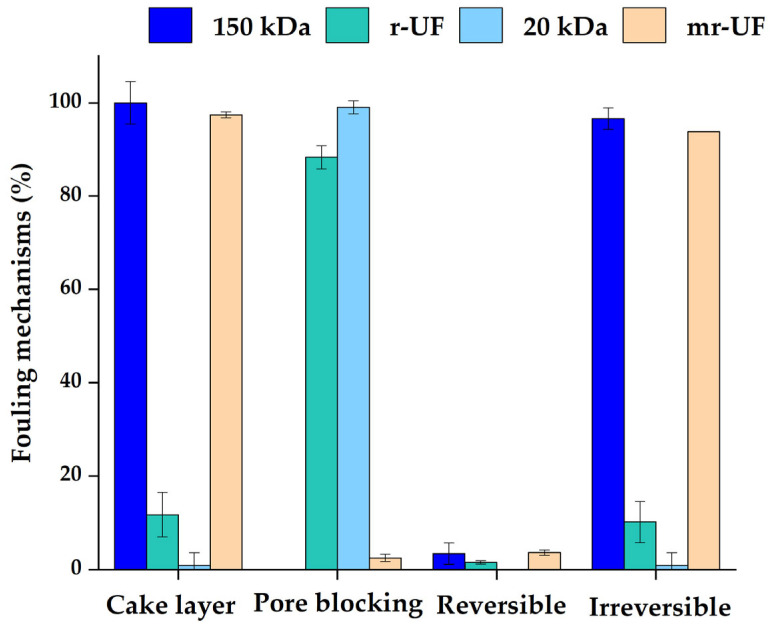
Membrane fouling resistance mechanisms.

According to fouling resistance analysis, the r-UF and mr-UF membranes presented lower fouling resistance due to fouling (Rf) (7.92 ± 0.57 × 10^12^ m^−1^ and 9.90 ± 0.1 × 10^12^ m^−1^, respectively), compared with the c-150 kDa and c-20 kDa membranes (1.56 ± 0.07 × 10^13^ m^−1^ and 1.25 ± 0.50E × 10^13^ m^−1^, respectively). This is most probably due to the higher surface roughness values of commercial membranes compared to recycled ones. As mentioned before, membrane fouling formation is a multifactorial process affected by several factors including membrane roughness [34,35]. Additionally, Figure 5 shows the different influential fouling mechanisms. Cake layer resistance includes reversible and irreversible cake layer resistance, while irreversible resistance values consist of both irreversible cake layer and pore blocking mechanisms. 

**Figure 6:**
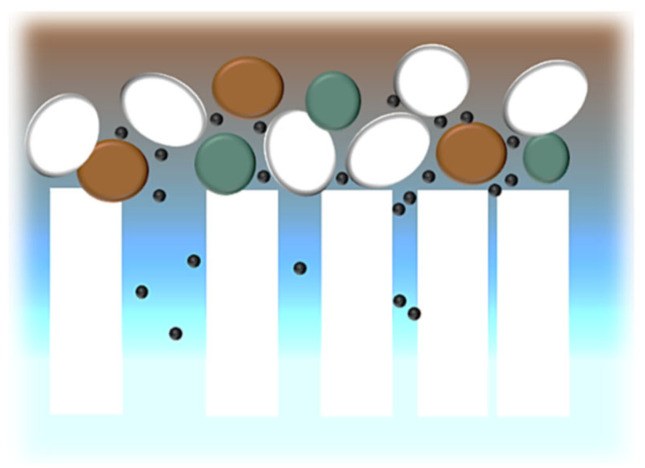
Cake layer deposition acting as a previous filter; adapted from [36].

For both membranes that presented lower MWCO (r-UF and c-20 kDa), the main mechanism affecting fouling was pore blocking. On the other hand, for membranes with higher MWCO (c-150 kDa and mr-UF membrane), the main mechanism was cake layer deposition. Even though it could be expected that pore blocking would be larger on membranes with higher MWCO, it could be that cake layer deposition itself happened faster than pore blocking, provoking the formation of a physical barrier on the membrane surface. Then, larger molecules that would have been participating in pore blocking would be retained by this barrier, with only the smaller molecules reaching pores (and even blocking the smaller ones) (Figure 6) [36].

**Figure 7:**
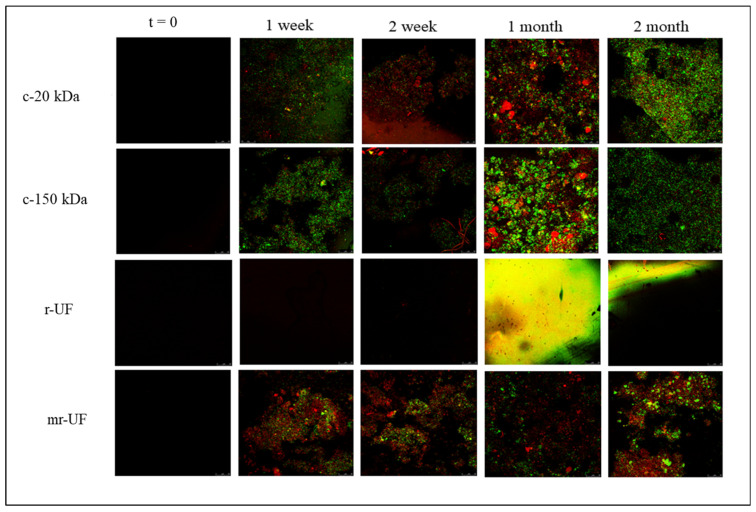
Confocal laser scanning microscopy (CLSM) images.

It is interesting to note that both the c-150 kDa and mr-UF membranes presented greater irreversible fouling. According to Le-Clech et al., irreversible fouling deposition over the membrane’s surface and interior pores more seriously affected membranes with larger-sized pores in the long term [36]. Additionally, it is important to know which type of fouling affected the membranes most. Even though membranes for water and wastewater treatment suffer from various types of fouling, biofouling is the main type of fouling affecting these membranes [37,38]. To check if membranes were affected by biofouling deposition during the experimental period, CLSM images were taken (Figure 7). 

**Figure 8:**
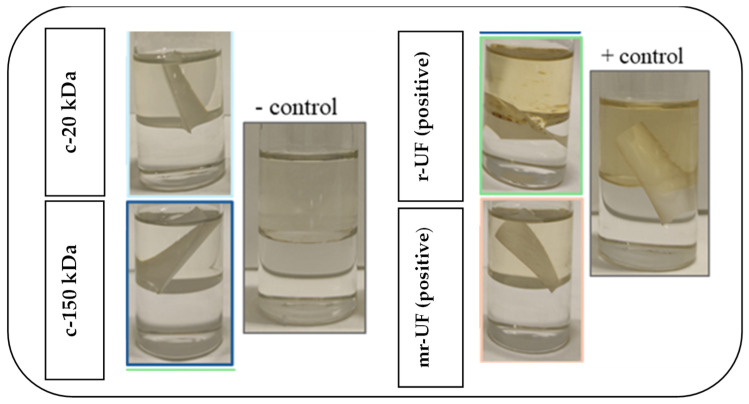
Fujiwara test results.

Confocal images show that both commercial membranes (c-150 kDa and c-20 kDa) present more attached biofouling, particularly at the late stages of the experimental period. On the other hand, the r-UF membrane, according to CLSM images, does not have any attached biofouling. This result is very promising because it clearly shows the antibiofouling character of the r-UF membrane. Moreover, the mr-UF membrane showed some degree of attached biofouling on the membrane surface that did not vary significantly during the experimental time, although, in general, it also presented less biofouling than the commercial membranes. Additionally, other works have already emphasized that r-UF membranes could be less prone to fouling due to contact with NaOCl during the recycling process [18,20]. Firstly, to confirm that the r-UF membrane was affected by halogenated compounds, a Fujiwara test was performed. The results are presented in Figure 8. As expected, both recycled membranes (r-UF and mr-UF) produced positive results.

Finally, an EDX analysis was conducted to study the chemical composition of the membranes’ surface and to ensure that chlorine is, in fact, present on the membrane surface (Table 3). The obtained results revealed that, as expected, chorine was present in higher amounts on recycled membranes. Specifically, the amount of chlorine in r-UF and mr-UF was around four times higher than in the commercial membranes (c-20 kDa and c-150 kDa). The presence of chlorine on the membrane surface is probably a consequence of the recycling process. The chlorinated compounds used in the recycling process may have changed the properties of the membrane’s surface, providing the recycled membrane with an antibiofouling character. The chlorine on the membrane surface might prevent the attachment of the bacteria found in the MBR system, ultimately reducing the biofouling phenomena on the membrane. This behavior on membranes that have chlorinated compounds on their surface has previously been observed [39].

On the other hand, the percentage of nitrogen in mr-UF is three times higher than in r-UF, which could be attributed to the nitrogen element of PEI used in the modification process, as observed in previous work [18]. Even though CA-PEI modification is intended to supply an antibiofouling character to membranes, the antibiofouling properties of the r-UF membrane itself have been shown to be even more effective. However, CA-PEI surface modification may have overlayed the chlorine compounds, reducing the antibiofouling properties of the mr-UF membrane compared to the r-UF membrane.

## 4. Conclusions

For the first time, an innovative long-term study to validate the use of r-UF membranes as aMBR submerged membranes has been conducted. The r-UF membrane and the mr-UF show excellent permeate quality that is comparable to different commercial UF membranes commonly used in MBR systems. Additionally, both the r-UF and mr-UF membranes showed a steadier pressure and less decline in permeability as the experimental period proceeded. The r-UF and mr-UF membranes also presented lower fouling resistance compared to commercial membranes. However, the more noteworthy result is that r-UF did not develop any biofouling during the experimental period (96 days) and presented the lowest fouling resistance value among all studied membranes. The present work also confirms that r-UF membrane surfaces contain chlorine that comes from the recycling process, granting the r-UF membrane its outstanding antibiofouling character. Therefore, this study shows that r-UF itself could be used on aMBR systems without the need for additional surface modification.

The results obtained regarding r-UF membranes are encouraging. Membranes’ acquisition and replacement costs, along with maintenance costs, represent a high percentage of expenses involved in membrane processes. Consequently, the results obtained in this study regarding r-UF membranes are encouraging, consolidating the r-UF membrane as a very promising alternative for use in aMBR systems. Moreover, the indirect recycling of end-of-life RO membranes could also be a valuable tool in confronting the challenges involved in waste management, helping to save on raw materials, energy consumption, and pollutant emission within environmental applications or industry processes in which membranes are used and therefore aligning with the principles of the circular economy.

## Figures and Tables

**Table 1:** Synthetic urban wastewater’s characteristics.

	Average	Standard Deviation
**EC (mS/cm)**	2.9	0.4
**COD (mg/L)**	421.4	23.1
**BOD_5_ (mg/L)**	226.3	17.4
**TOC (mg/L)**	132.5	9.6
**TN (mg/L)**	31.4	2.0
**TP (mg/L)**	5.0	0.9

**Table 2:** Membranes’ technical data.

		c-150 kDa	r-UF	c-20 kDa	mr-UF
**Material**		Polyethersulfone (PES)	Polyethersulfone (PES)	Polyethersulfone (PES)	Polyethersulfone (PES)
**Backing material**		Polypropylene (PP)	Polyethylene terephthalate (PET)	Polypropylene (PP)	Polyethylene terephthalate (PET)
**Permeability** **(L·m^−2^·h^−1^ bar^−1^)**		566.17 ± 9.39	39.03 ± 1.74	56.97 ± 1.55	192.68 ± 1.57
**M.W.C.O (kDa)**		87.64 ± 0.65	33.76 ± 4.16	24.63 ± 3.82	85.04 ± 6.87
**Contact angle**		58.00 ± 0.99	59.01 ± 1.25	57.05 ± 1.25	59.21 ± 1.76
**Roughness**	**R_a_ (nm)**	8.4 ± 2.6	4.7 ± 0.6	11.7 ± 3.7	5.2 ± 0.9
	**R_q_ (nm)**	6.6 ± 2.2	6.3 ± 1.2	8.9 ± 2.7	7.5 ± 1.3

**Table 3:** Chemical composition analysis by EDX.

	c-150 kDa		r-UF [17]		c-20 kDa		mr-UF [17]	
	% Weight	% Atomic	% Weight	% Atomic	% Weight	% Atomic	% Weight	% Atomic
**C**	59.12	68.69	69.75	77.51	59.74	69.25	64.25	71.94
**N**	2.91	2.90	1.32	1.26	2.84	2.82	3.57	3.34
**O**	27.18	23.70	21.94	18.30	26.79	23.31	26.47	22.25
**S**	10.48	4.56	5.97	2.49	10.52	4.57	5.23	2.19
**Cl**	0.13	0.05	0.42	0.16	0.11	0.04	0.49	0.18

## Data Availability

The original contributions presented in the study are included in the article, further inquiries can be directed to the corresponding authors.
